# Postoperative complications observed with robotic versus laparoscopic surgery for the treatment of rectal cancer: An updated meta-analysis of recently published studies

**DOI:** 10.1097/MD.0000000000027158

**Published:** 2021-09-10

**Authors:** Chengkui Liu, Xiaoqing Li, Qingfeng Wang

**Affiliations:** a Department of Gastrointestinal Surgery, Zibo Central Hospital, Zibo, Shandong, PR China; b Operating Room, Zibo Central Hospital, Zibo, Shandong, PR China.

**Keywords:** laparoscopic surgery, postoperative complications, rectal cancer, robotic surgery

## Abstract

**Background:**

This is an updated meta-analysis comparing the postoperative complications observed with robotic versus laparoscopic surgery (LS) for the treatment of rectal cancer.

**Methods:**

Cochrane central, MEDLNE (Medical Literature Analysis and Retrieval System Online), EMBASE (Excerpta Medica dataBASE), Google Scholar, Web of Science and http://www.ClinicalTrials.gov were searched for studies (published after the year 2015), comparing robotic versus LS for the treatment of rectal cancer. The postoperative outcomes were considered as the endpoints in this analysis. RevMan 5.4 was used to carry out the statistical analysis. Risk ratio (RR) with 95% confidence intervals (CI) were used to represent the results following data analysis.

**Results:**

A total number of 22,744 participants were included in this study whereby 9178 participants were assigned to the robotic surgery and 13,566 participants were assigned to the LS group. The time period of patients’ enrollment varied from years 2007 to 2017. Our results showed that overall complications (RR: 0.91, 95% CI: 0.71–1.17; *P* = .45), wound complications (RR: 0.81, 95% CI: 0.64–1.04; *P* = .09), anastomotic leak (RR: 1.12, 95% CI: 0.88–1.42; *P* = .37), anastomotic bleeding (RR: 0.88, 95% CI: 0.29–2.64; *P* = .82), stoma-related complications (RR: 0.88, 95% CI: 0.24–3.21; *P* = .85), intra-abdominal abscess (RR: 0.53. 95% CI: 0.22–1.31; *P* = .17), urinary tract infection (RR: 0.94, 95% CI: 0.53–1.66; *P* = .83), enterocolitis (RR: 1.35, 95% CI: 0.38–4.71; *P* = .64), reoperation (RR: 0.85, 95% CI: 0.46–1.54; *P* = .58), and mortality (RR: 0.75, 95% CI: 0.34–1.62; *P* = .46) were not significantly different between robotic-assisted versus LS for rectal cancer. Postoperative ileus (RR: 1.21, 95% CI: 0.81–1.81; *P* = .34), readmission (RR: 1.17, 95% CI: 0.75–1.83; *P* = .48), and urinary retention (RR: 0.51, 95% CI: 0.21–1.23; *P* = .14) were also similarly manifested.

**Conclusions:**

In this updated meta-analysis, both robotic and laparoscopic surgeries were equally effective for the treatment of rectal cancer. Similar postoperative complications were observed. However, our analysis was restricted only to postoperative outcomes, parameters such as duration of surgery were not taken into consideration.

## 1 Introduction

Today, colorectal cancer, including approximately 30% of cases of rectal cancer, is rapidly increasing.^[1]^ A family history of colorectal cancer, an advanced age, consumption of high amount of red meat, food products high in fats, low calcium and low fiber, pre-existing irritable bowel diseases, obesity, rectal polyps are all risk factors for the development of colorectal cancer.^[2]^ This disease is life-threatening, and if regular health check-ups are not done, colorectal carcinoma might have reached an advanced stage when discovered. If detected earlier, surgery could prevent the spread of colorectal cancer.^[3]^ With progress in technology as well as in operative techniques, laparoscopic surgery (LS)^[4]^ and most recently robotic assisted^[5]^ surgery have now become the possible treatment strategies for colorectal carcinoma. Several meta-analyses and systematic reviews have been carried out to compare laparoscopic versus robotic assisted surgeries for the treatment of rectal cancers^[6,7]^ However, shortcomings were observed. In one analysis, the authors stated that the quality of the evidence was moderate for most outcomes.^[8]^ In an overview of systematic reviews with quality assessment of current evidence, the authors concluded that high-quality systematic reviews in which selection of high quality studies and combined with adequate methodology would be needed to clarify the true efficacy of robotic surgery.^[9]^ Nevertheless, with further improvement in more sophisticated equipment and modern tools which are being used today, we aimed to include only recently published studies (after the year 2015), to compare the postoperative complications (POC) observed with robotic versus LS for the treatment of rectal cancer.

## 2 Methods

### 2.1 Data sources

Cochrane central, MEDLNE (Medical Literature Analysis and Retrieval System Online), EMBASE (Excerpta Medica dataBASE), Google Scholar, Web of Science and http://www.ClinicalTrials.gov were searched for publications comparing robotic versus LS for the treatment of rectal cancer.

### 2.2 Search terms and searched strategies

English-based publications were considered relevant in this analysis. The following searched terms or phrases were used:

(a) Robotic, laparoscopic, rectal cancer;
(b) Robotic, laparoscopic, rectal carcinoma;
(c) Robotic surgery, LS, rectal cancer;
(d) Rectal cancer and surgery;
(e) Rectal carcinoma and surgery.

Not a single abbreviation was required to be used in the search process.

### 2.3 Inclusion and exclusion criteria

The inclusion criteria were:

(a) Studies that compared robotic versus LS for the treatment of rectal cancer;
(b) Studies that were published after the year 2015;
(c) Studies that reported POC;
(d) Studies that consisted of dichotomous data;
(e) Studies which were published in English language.

The exclusion criteria were:

(a) studies that did not compare robotic versus LS for the treatment of rectal cancer;
(b) studies that compared robotic versus LS for the treatment of rectal cancer but were published before or during the year 2015;
(c) non-English publications;
(d) studies that did not report postoperative outcomes;
(e) studies that were repeatedly found in different search databases.

### 2.4 Outcomes and follow-UPS

The outcomes which were reported in the original studies have been listed in Table 1.

**Table 1:** Outcomes reported.

Studies	Outcomes	Follow-up time periods
Bo 2019^[13]^	Wound complication, UTI, urinary retention, stress ulcer, pulmonary, liver dysfunction, intraluminal bleeding, intra-abdominal abscess, intestinal fistula, injury of urinary system, infection via catheter, ileus, cerebrovascular, enterocolitis, cardiac, anastomosis bleeding, anastomosis leakage	30 days postoperative
Chen 2017^[14]^	Wound complication, acute myocardial infarction, pulmonary collapse, acute respiratory failure, general surgical complications, paralytic ileus, peritonitis and retroperitoneal infections, hemoperitoneum, postoperative infection	5 years period
Colombo 2016^[15]^	Anastomotic leakage, ischemic colitis, postoperative ileus, peri-anastomotic abscess, early postoperative surgery, mortality due to cancer	15–35 months
Feroci 2016^[16]^	Anastomotic leak, peritoneal hemorrhage, stomal stricture, wound infection, ileus, abdominal pain, reoperation, mortality for rectal cancer	37.4 months
Galata 2019^[17]^	Reoperation, anastomotic leak, postoperative ileus, intra-abdominal abscess, readmission, other complications, surgical site infection	12 months
Garfinkle 2019^[18]^	Superficial surgical site infection, deep surgical site infection, wound dehiscence, anastomotic leak, postoperative ileus, pneumonia, urinary tract infection, pulmonary embolism, deep vein thrombosis, acute renal failure, stroke, cardiac arrest, sepsis, septic shock, reoperation, mortality, readmission	30 days
Hopkins 2019^[19]^	30 days mortality, 90 days mortality, readmission	3 months
Lelpo 2017^[20]^	Overall complications, readmission, anastomotic leakage, intra-abdominal abscess	5 years
Jayne 2017^[21]^	Overall postoperative complications, mortality within 30 days and above 30 days, gastrointestinal complication, urinary complication, surgical site infection, respiratory complication, cardiac complication, cerebrovascular complication, anastomotic leakage	6 months
Kim 2016^[22]^	Anastomotic leakage, reoperation	
Kim 2017A^[23]^	Anastomotic leakage, ileus, acute voiding difficulty, stoma-related complication, wound discharge, bleeding	
Kim 2017B^[24]^	Anastomotic leakage, wound infection, ileus, bleeding, postoperative mortality	5 years
Law 2016^[25]^	Overall complications, 30-day mortality, reoperation, anastomotic leak, ileus, wound complication, urine retention, deep vein thrombosis, cardiac complication	30 days
Liu 2019^[26]^	Wound infection, urinary tract infection, urinary retention, anastomotic leak, anastomotic bleeding, small bowel obstruction	30 days
Shiomi 2019^[27]^	Anastomotic leakage, anastomotic bleeding, prolonged ileus, urinary retention, urinary tract infection, wound infection, pelvic abscess, enterocolitis, pneumonia	-

The postoperative outcomes which were assessed included:

(a) overall complications;
(b) wound complication;
(c) anastomotic leak;
(d) anastomotic bleeding;
(e) stoma-related complication;
(f) postoperative ileus;
(g) intra-abdominal abscess;
(h) urinary retention;
(i) enterocolitis;
(j) urinary tract infection;
(k) readmission;
(l) reoperation;
(m) mortality.

### 2.5 Data extraction and quality assessment

The authors independently extracted data from the original studies. Data including the surname of the first authors of each paper, the year of publication, the POC which were reported, the total number of participants who were treated with robotic and laparoscopic surgeries respectively, the methodological quality of the studies, the follow-up time period, the baseline features including age, percentage of male participants, and the body mass index of the participants, the types of study, the patients’ enrollment time period, and the number of events were carefully extracted.

Any disagreement which followed were carefully discussed and resolved among the authors.

The methodological quality of the studies were assessed by the Cochrane tool for the randomized trials^[10]^ and by the Newcastle Ottawa Scale (NOS)^[11]^ for the observational studies. A grade ranging from A to C was allotted, grade A representing a low risk of bias whereas grade C represented a high risk of bias.

### 2.6 Statistical analysis

This is a meta-analysis including data which were extracted from previously published studies. RevMan 5.4 was used to carry out the statistical analysis. Heterogeneity was assessed by 2 simple statistical test: the *Q* statistic test and the *I*^2^ statistic test. A *P* value ≤.05 was considered statistically significant. A result with a *P* value >.05 was considered insignificant statistically. For the *I*^2^ test, the lower the *I*^2^ value, the lower the heterogeneity. If an *I*^2^ value of <50% was obtained, a fixed effect model was used or else, a random statistical model was used during the data analysis. Risk ratio (RR) with 95% confidence intervals (CI) were used to represent the results following data analysis.

Sensitivity analysis was also carried out. An exclusion method was used to carry sensitivity analysis. Each study was excluded one by one, and an analysis was carried out each time excluding that particular study. The results which were obtained were compared with the main results of this analysis for any significant change.

Publication bias was also assessed by visually observing funnel plots for any asymmetry.

### 2.7 Ethical approval

This study is a meta-analysis of previously published studies and therefore, an ethical approval was not required.

## 3 Results

### 3.1 Search outcomes

The preferred reporting items for systematic reviews and meta-analyses (PRISMA) guideline was followed.^[12]^ A total number of 1276 publications were obtained. Following an initial assessment after carefully studying the abstracts and titles, 895 publications were excluded. Two hundred and fifty five (255) repeated studies were further eliminated among the remaining publications. One hundred and twenty six (126) full text articles were assessed for eligibility. Based on the inclusion and exclusion criteria, further eliminations were carried due to the following reasons:

- relevant studies which were published on or before the year 2015 (n = 34),
- non-English publications (n = 7);
- systematic reviews and meta-analyses or literature reviews (42);
- case studies (n = 28).

**Figure 1:**
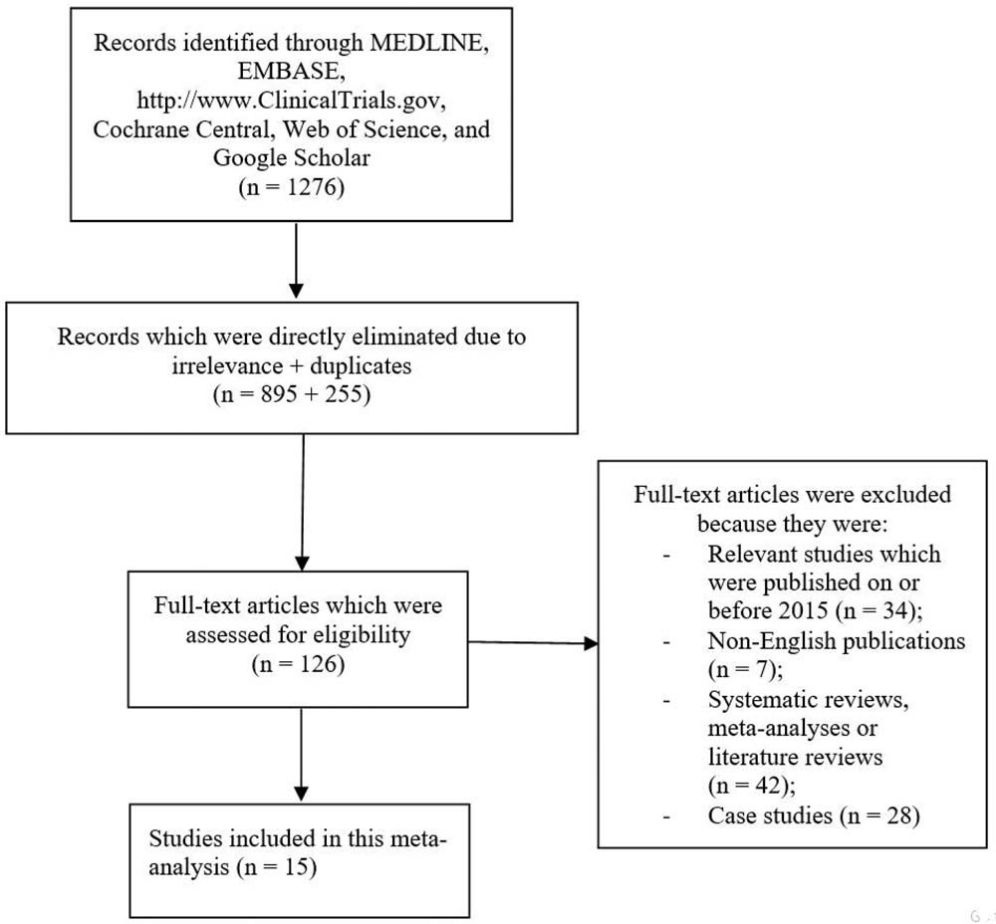
Flow diagram showing the study selection.

Finally, only 15 studies^[13–27]^ were included in this analysis as shown in Fig. 1.

### 3.2 General and baseline features

The main features of the studies have been listed in Table 2. Two studies were randomized trials whereas the remaining 13 studies were observational studies. A total number of 22,744 participants were included in this study whereby 9178 participants were assigned to the robotic surgery and 13,566 participants were assigned to the LS groups as shown in Table 2. The time period of patients’ enrollment varied from years 2007 to 2017.

**Table 2:** Main features of the studies.

Studies	Type of study	Year of patients enrollment	No. of participants assigned to robotic assisted surgery (n)	No. of participants assigned to laparoscopic assisted surgery (n)	Bias risk grade
Bo 2019	Retrospective	2010–2016	556	1139	B
Chen 2017	Retrospective	2008–2012	4744	5578	B
Colombo 2016	OS	2009–2013	60	60	B
Feroci 2016	Retrospective	2008–2014	53	58	B
Galata 2019	Prospective	2016–2017	18	33	B
Garfinkle 2019	Prospective	2016	154	213	B
Hopkins 2019	Prospective	2010–2014	2472	5144	B
Lelpo 2017	OS	2010–2017	86	112	B
Jayne 2017	Randomized trial	2011–2014	237	234	B
Kim 2016	Prospective	2010–2012	33	66	B
Kim 2017A	Randomized trial	2012–2015	66	73	B
Kim 2017B	Retrospective	2007–2014	272	460	B
Law 2016	Prospective	2008–2015	220	171	B
Liu 2019	Retrospective	2015–2017	80	116	B
Shiomi 2019	Retrospective	2010–2015	127	109	B
Total no of patients (n)			9178	13,566	

Following a methodological assessment, a grade B was allotted to the studies representing a moderate risk of bias.

The baseline features of the participants have been listed in Table 3. According to Table 3, the mean age of the participants from the robotic surgery group varied from 57.0 to 66.0 years and the mean age of those participants who were assigned to the laparoscopic group varied from 58.0 to 68.0 years. The percentage of male participants from the robotic group varied from 50.9% to 77.3%, whereas the percentage of male participants who were assigned to the laparoscopic group varied from 56.7% to 72.4% as shown in Table 3. The value for body mass index was also given in the table.

**Table 3:** Baseline features of the participants.

Studies	Age, yr	Males (%)	BMI, kg/m^2^
	RS/LS	RS/LS	RS/LS
Bo 2019	57.0/58.0	62.4/62.2	23.3/23.0
Chen 2017	–	–	–
Colombo 2016	62.0/60.0	66.7/70.0	25.8/23.8
Feroci 2016	66.0/66.0	50.9/72.4	24.6/24.6
Galata 2019	60.0/62.3	–	26.0/27.4
Garfinkle 2019	61.9/63.8	68.8/59.6	28.0/27.3
Hopkins 2019	59.0/59.0	66.0/62.0	–
Lelpo 2017	63.9/61.6	55.8/59.8	26.1/25.7
Jayne 2017	64.4/65.5	67.9/67.9	–
Kim 2016	57.0/58.2	69.7/69.7	23.2/23.3
Kim 2017A	60.4/59.7	77.3/71.2	24.1/23.6
Kim 2017B	59.2/63.9	68.0/64.3	23.5/23.3
Law 2016	65.0/67.0	67.3/56.7	24.9/24.6
Liu 2019	62.0/59.7	66.3/62.1	23.1/23.3
Shiomi 2019	65.0/68.0	73.2/59.6	23.7/22.8

### 3.3 Main results of this analysis

**Figure 2:**
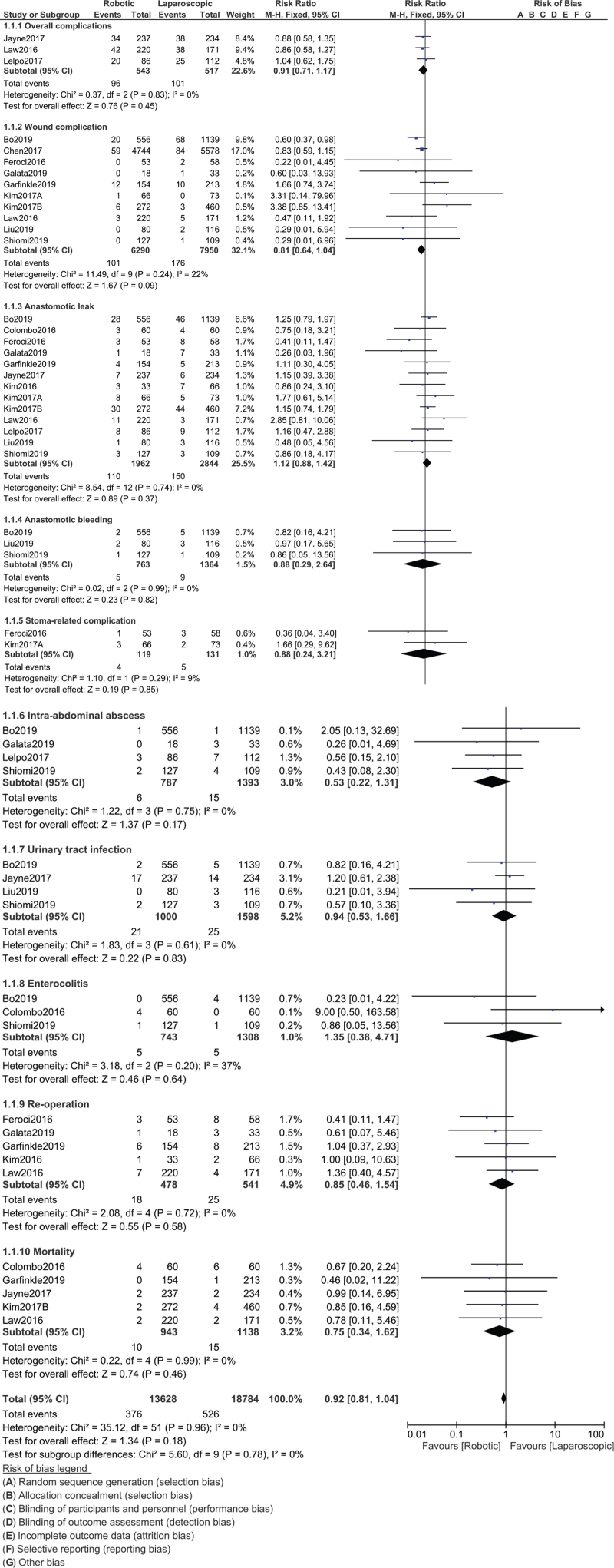
Postoperative outcomes observed with robotic versus laparoscopic surgery for the treatment of rectal cancer (Part A).

**Figure 3:**
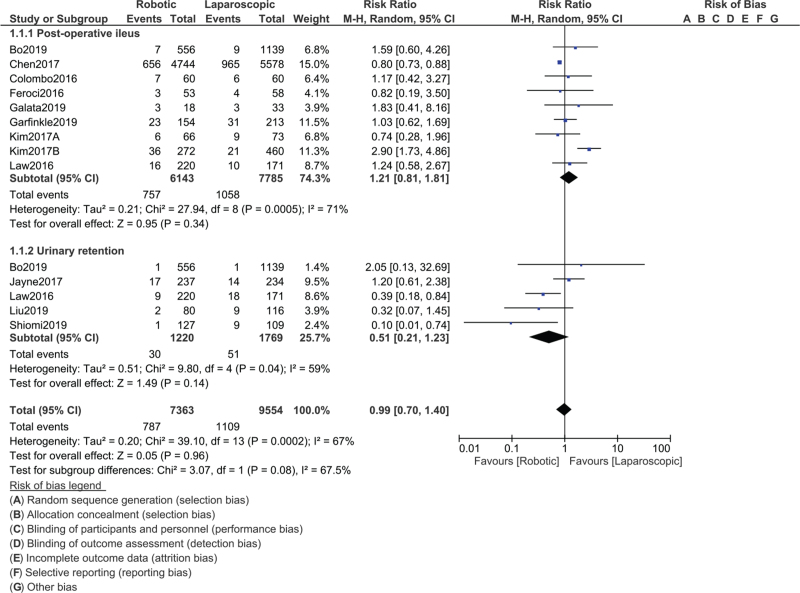
Postoperative outcomes observed with robotic versus laparoscopic surgery for the treatment of rectal cancer (Part B).

**Figure 4:**
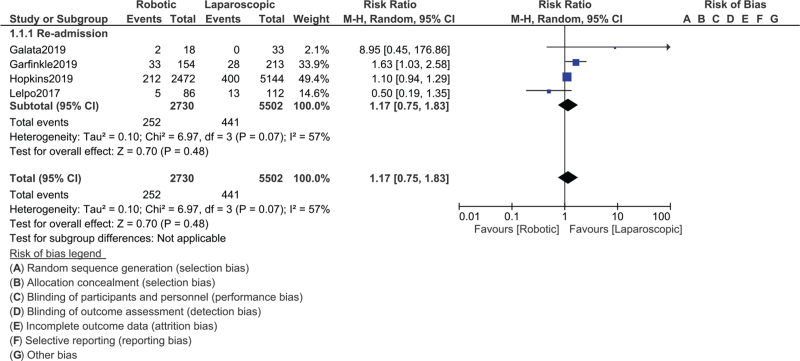
Readmission observed with robotic versus laparoscopic surgery for the treatment of rectal cancer.

Our results showed that overall complications (RR: 0.91, 95% CI: 0.71–1.17; *P* = .45), wound complications (RR: 0.81, 95% CI: 0.64–1.04; *P* = .09), anastomotic leak (RR: 1.12, 95% CI: 0.88–1.42; *P* = .37), anastomotic bleeding (RR: 0.88, 95% CI: 0.29–2.64; *P* = .82), stoma-related complications (RR: 0.88, 95% CI: 0.24–3.21; *P* = .85), intra-abdominal abscess (RR: 0.53, 95% CI: 0.22–1.31; *P* = .17), urinary tract infection (RR: 0.94, 95% CI: 0.53–1.66; *P* = .83), enterocolitis (RR: 1.35, 95% CI: 0.38–4.71; *P* = .64), reoperation (RR: 0.85, 95% CI: 0.46–1.54; *P* = .58), mortality (RR: 0.75, 95% CI: 0.34–1.62; *P* = .46) were not significantly different between robotic-assisted versus LS for rectal cancer as shown in Fig. 2. Postoperative ileus (RR: 1.21, 95% CI: 0.81–1.81; *P* = .34) and urinary retention (RR: 0.51, 95% CI: 0.21–1.23; *P* = .14) were also similarly manifested as shown in Fig. 3. In addition, there was also no significant difference in readmission (RR: 1.17, 95% CI: 0.75–1.83; *P* = .48) among patients who were treated with robotic versus LS as shown in Fig. 4.

The results were summarized in Table 4.

**Table 4:** Results of this analysis.

Outcomes which were assessed	RR with 95% CI	*P* value	*I*^2^ value (%)
Overall complications	0.91 [0.71–1.17]	.45	0
Wound complication	0.81 [0.64–1.04]	.09	22
Anastomotic leak	1.12 [0.88–1.42]	.37	0
Anastomotic bleeding	0.88 [0.29–2.64]	.82	0
Stoma-related complication	0.88 [0.24–3.21]	.85	9
Post-operative ileus	1.21 [0.81–1.81]	.34	71
Intra-abdominal abscess	0.53 [0.22–1.31]	.17	0
Urinary retention	0.51 [0.21–1.23]	.14	59
Urinary tract infection	0.94 [0.53–1.66]	.83	0
Enterocolitis	1.35 [0.38–4.71]	.64	37
Re-admission	1.17 [0.75–1.83]	.48	57
Re-operation	0.85 [0.46–1.54]	.58	0
Mortality	0.75 [0.34–1.62]	.46	0

**Figure 5:**
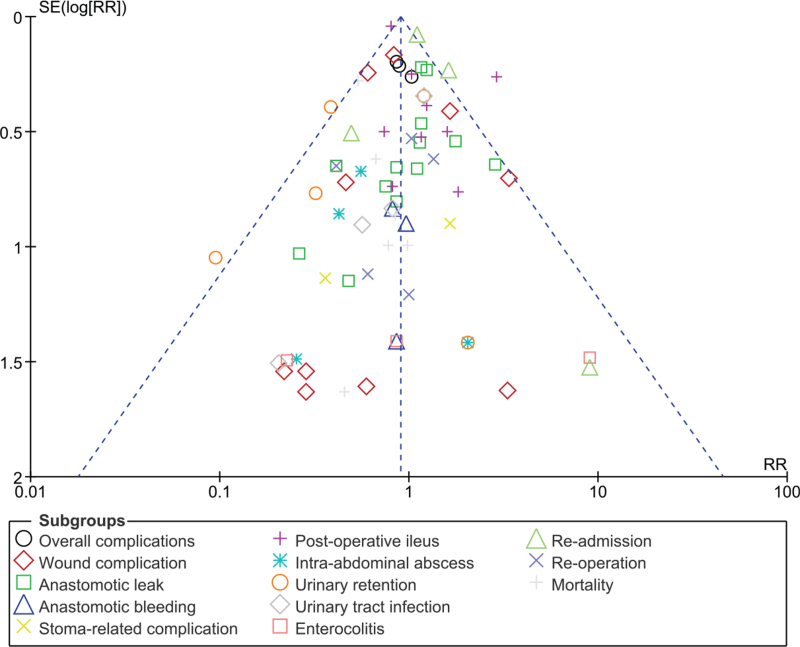
Funnel plot showing the assessment of publication bias.

Consistent results were obtained throughout. There was no evidence of publication bias when visually assessing the funnel plot which was represented by Fig. 5.

## 4 Discussion

Using the most recent data (studies published after the year 2015), we aimed to compare the POC observed with robotic versus LS for the treatment of rectal cancer.

Our results did not show any significant difference in POC between robotic and LS for the treatment of rectal carcinoma. A similar risk of overall complications, including anastomotic leak and bleeding, intra-abdominal abscess, stoma-related complications, urinary tract infections, urinary retention, enterocolitis, readmission, reoperation, and mortality was observed between these two categories of treatment.

Another meta-analysis focusing on rectal cancer showed POC to be similar with both robotic and LS.^[28]^ It should be noted that the analysis consisted of 16 studies which were published before July 2011 whereas our current analysis included 15 studies which were published after the year 2015.

A meta-analysis which was published in the year 2015, and including 17 studies with 2224 participants showed robotic assisted surgery to be a good alternative to LS and the authors also showed that robotic surgery could enhance postoperative recovery in patients with rectal cancer, and had better recovery in voiding and sexual function.^[29]^

Nevertheless, another meta-analysis published in the year 2017, comparing robotic-assisted versus conventional laparoscopic operation in anus-preserving rectal cancer showed the Da Vinci robot to be better compared with LS in terms of POC, blood loss, hospital stay, conversion to open surgery when compared with the LS.^[30]^ However, LS had an advantage in terms of operative time, but since our analysis was only specifically based on postoperative outcomes, we did not compare the duration of surgery, conversion to open surgery and hospital stay.

In an observational study of patients outcomes from a district general hospital with over a decade of experience with robotic rectal cancer surgery, where 337 patients were included, the authors demonstrated how the selective use of robotic surgery by a group of rectal cancer team could help to decrease the rate of rectal cancer recurrence as well as decrease permanent stoma rates.^[31]^

Our current analysis only focused on the postoperative outcomes observed with robotic versus LS for rectal cancer. We did not assess the duration of surgery or the conversion of invasive to open surgery.^[8]^ Nevertheless, based on previously published studies, it was found that robotic assisted surgery was better in terms of less blood loss, less conversion to open surgery, but it was associated with a longer duration of surgery compared with the conventional LS.^[32]^

Micro Hand S, a Chinese surgical robot, was recently introduced clinically, to be used as a novel robotic platform. When this robotic approach surgical procedure was compared with other conventional approaches, the total rate of surgical success was similar.^[33]^ The authors concluded that this robotic surgical approach was feasible and safe to be used showing comparable postoperative outcomes, but with superiority in blood loss, bowel function recovery, and length of hospital stay. However, the operative time was longer with the robotic approach when compared with the other conventional approaches. It is also believed that as costs and operating time decline with robotic surgery in the future as technology progresses, robotic surgery might even replace the traditional laparoscopic techniques one day.^[34]^

However, even though many published studies have shown the clinical benefits of robotic surgery for patients with rectal carcinoma, critically low quality evidence suggests that robotic-assisted surgery for rectal cancers decreased the likelihood of conversion to open surgery, but other clinical benefits remain unclear.^[9]^ The authors finally requested high qualities systematic reviews to clarify this issue.

### 4.1 Limitations

This study also has limitations. First of all, only a few outcomes were common in almost all the studies. Therefore, we could not include all the studies when assessing each of the outcomes. Secondly, the follow up time period was not same in all the studies. This could have affected the results of this analysis. Another limitation was the fact that data which were extracted from randomized trials and observational studies combined and analyzed. This might have had an impact on the final results. Also, our analysis is an updated version including only studies which were published after the year 2015. This could also have an impact on the final outcome of this analysis and this study might only be a subtype of previously published meta-analyses. Moreover, the length period of disease was ignored in this analysis. It might be possible that the cause of mortality was related to the cancer itself instead to the surgery. Therefore, this could be also another limitation when assessing for mortality.

## 5 Conclusions

In this updated meta-analysis, both robotic and LS were equally effective for the treatment of rectal cancer. Similar POC were observed. However, our analysis was restricted only to postoperative outcomes, parameters such as duration of surgery were not taken into consideration.

## Author contributions

The authors Chengkui Liu, Xiaoqing Li, and Qingfeng Wang were responsible for the conception and design, acquisition of data, analysis and interpretation of data, drafting the initial manuscript and revising it critically for important intellectual content. All the authors agreed and approved the manuscript as it is.

**Conceptualization:** Chengkui Liu, Xiaoqing Li, Qingfeng Wang.

**Data curation:** Chengkui Liu, Xiaoqing Li, Qingfeng Wang.

**Formal analysis:** Chengkui Liu, Xiaoqing Li, Qingfeng Wang.

**Funding acquisition:** Chengkui Liu, Xiaoqing Li, Qingfeng Wang.

**Investigation:** Chengkui Liu, Xiaoqing Li, Qingfeng Wang.

**Methodology:** Chengkui Liu, Xiaoqing Li, Qingfeng Wang.

**Project administration:** Chengkui Liu, Xiaoqing Li, Qingfeng Wang.

**Resources:** Chengkui Liu, Xiaoqing Li, Qingfeng Wang.

**Software:** Chengkui Liu, Xiaoqing Li, Qingfeng Wang.

**Supervision:** Chengkui Liu, Xiaoqing Li, Qingfeng Wang.

**Validation:** Chengkui Liu, Xiaoqing Li, Qingfeng Wang.

**Visualization:** Chengkui Liu, Xiaoqing Li, Qingfeng Wang.

**Writing – original draft:** Chengkui Liu, Xiaoqing Li, Qingfeng Wang.

**Writing – review & editing:** Chengkui Liu, Xiaoqing Li, Qingfeng Wang.
